# Natural Phytochemicals, Luteolin and Isoginkgetin, Inhibit 3C Protease and Infection of FMDV, In Silico and In Vitro

**DOI:** 10.3390/v13112118

**Published:** 2021-10-21

**Authors:** Sirin Theerawatanasirikul, Nattarat Thangthamniyom, Chih-Jung Kuo, Ploypailin Semkum, Nantawan Phecharat, Penpitcha Chankeeree, Porntippa Lekcharoensuk

**Affiliations:** 1 Department of Anatomy, Faculty of Veterinary Medicine, Kasetsart University, Bangkok 10900, Thailand; fvetsrth@ku.ac.th; 2 Department of Microbiology and Immunology, Faculty of Veterinary Medicine, Kasetsart University, Bangkok 10900, Thailand; Golf_Zealand@hotmail.com (N.T.); ploypailinvet69@gmail.com (P.S.); nantawan_ph@hotmail.com (N.P.); penpitchac56@nu.ac.th (P.C.); 3 Department of Veterinary Medicine, National Chung Hsing University, Taichung 40227, Taiwan; 4 Center for Advanced Studies in Agriculture and Food, Kasetsart University Institute for Advanced Studies, Kasetsart University, Bangkok 10900, Thailand

**Keywords:** foot-and-mouth-disease virus (FMDV), luteolin, isoginkgetin, phytochemicals, FMDV 3C^pro^, antiviral activity

## Abstract

Foot-and-mouth-disease virus (FMDV) is a picornavirus that causes a highly contagious disease of cloven-hoofed animals resulting in economic losses worldwide. The 3C protease (3C^pro^) is the main protease essential in the picornavirus life cycle, which is an attractive antiviral target. Here, we used computer-aided virtual screening to filter potential anti-FMDV agents from the natural phytochemical compound libraries. The top 23 filtered compounds were examined for anti-FMDV activities by a cell-based assay, two of which possessed antiviral effects. In the *viral* and *post-viral entry* experiments, luteolin and isoginkgetin could significantly block FMDV growth with low 50% effective concentrations (EC50). Moreover, these flavonoids could reduce the viral load as determined by RT-qPCR. However, their prophylactic activities were less effective. Both the cell-based and the fluorescence resonance energy transfer (FRET)-based protease assays confirmed that isoginkgetin was a potent FMDV 3C^pro^ inhibitor with a 50% inhibition concentration (IC50) of 39.03 ± 0.05 and 65.3 ± 1.7 μM, respectively, whereas luteolin was less effective. Analyses of the protein–ligand interactions revealed that both compounds fit in the substrate-binding pocket and reacted to the key enzymatic residues of the 3C^pro^. Our findings suggested that luteolin and isoginkgetin are promising antiviral agents for FMDV and other picornaviruses.

## 1. Introduction

Foot-and-mouth-disease virus (FMDV) causes a severe and highly contagious transboundary disease of cloven-hoofed animals, which has a significant impact on livestock production worldwide [1,2]. FMDV belongs to the genus *Aphthovirus* in family *Picornaviridae*, order *Picornavirales* [3,4]. They consist of seven serotypes (A, O, C, Asia1, South African Territories (SAT) 1, SAT2, and SAT3), which are endemic in several countries [1,2,5]. Clinical signs of FMD comprise mild to severe blisters or vesicles on the mouth, nose, lips, and skin above hooves and toes, which result in severe production losses. The affected animals feel fever, extreme pain, and lameness. After recovery, FMDV may persist in asymptomatic animals. Hence, reducing FMDV circulating in endemic countries requires restricted movement of infected animals as well as contaminated people and animal products. As a result, preemptive culling of the FMDV-affected animals is recommended [2,6,7].

Although FMDV vaccines can stop some FMD outbreaks, vaccination does not prevent infection and takes several days to weeks to elicit an immune response against FMDV [7]. Moreover, matching the virus vaccine to the diverse field FMDV strains may be problematic. To effectively cope with FMD outbreaks and viral persistence, the ideal antiviral therapeutics should be immediately and broadly active on a large diversity of virus strains and serotypes to reduce clinical signs and viral shedding during emergency usage or the pre-vaccine-induced immunity period. Currently, there is no effective treatment available for FMDV; thus, antiviral agents for rapid prevention and control of FMD spread are still necessitated.

FMDV is a small non-enveloped virus with positive-sense, single-stranded RNA. FMDV is classified in the family *Picornaviridae* together with poliovirus (PV), human rhinovirus (HRV), enterovirus 71 (EV71), coxsackievirus B (CVB), and hepatitis A virus (HAV) [4]. The genome of FMDV encodes a single long open reading frame (ORF) of about 7 kb with two alternative initiation sites [3,8,9,10]. The 3C protease (3C^pro^), a virus-encoded protease, is the key enzyme for viral polyprotein processing, which is crucial for the viral life cycle. FMDV 3C^pro^ is a chymotrypsin-like cysteine protease, which is required for the 10 of 13 cleavages of the single polyprotein into intermediate precursors and final individual structural and nonstructural proteins [11,12]. In addition, FMDV 3C^pro^ is also involved in host cellular transcription and translation [3,13,14]. The essential roles of 3C^pro^ makes it an attractive antiviral target for use in combination with other measures for FMDV prevention and control. 

FMDV 3C^pro^ possesses cysteine protease-like characteristics structurally formed by folding of the polypeptides to bring Cys163, His46, Asp84, and Cys142 in proximity, resulting in a Cys-His-Asp catalytic triad and Cys142 flap [9,11,12,14]. In the catalytic process, the charge relay system exploits bridging charged amino acids (His and Asp) and nucleophile (thiol group of Cys) to attack the viral polyprotein substrate more actively. Interfering with this system could reduce the proteolytic function, although the catalytic residues are not dissociated. Antiviral drugs targeting the protease of picornaviruses have been developed. For example, rupintrivir (AG7088), an HRV 3C protease (3C^pro^) inhibitor, showed high antiviral activity in cultured cells. However, rupintrivir was not significantly effective against natural HRV infection [15,16,17].

Increasing evidence-based research has been conducted on the antiviral activities of herbal medicinal and purified natural phytochemical compounds, which demonstrated potential therapeutic benefits. Moreover, these phytochemicals are safe and non-cytotoxic to the host cells, which could be used as alternative medicines or combined with existing treatments [18,19]. The active substances from natural sources may be applied for antiviral drug design based on their structures. Among phytochemicals, flavonoids are a large group of plant polyphenolic compounds with various properties, such as anti-inflammatory, antioxidant, anticancer, antimicrobial, and antiviral activities [18,20]. Several studies have reported that flavonoids could inhibit picornaviruses, such as EV 71 [21,22], CVB [23,24,25], and PV [26]. The putative modes of their action have been reported, for example, counteracting against 3C^pro^ activity [27,28]. However, the exact mechanisms of flavonoids at the molecular level are still unclear. 

Toward drug discovery research, virtual screening is an effective computer-aided platform that can accelerate the search for new candidate antiviral agents in silico. This approach can be combined with virological and molecular biological techniques to reveal novel mechanisms of compounds on viral proteins based on structural relationships. In the current study, we utilized computer-aided virtual screening to select potential phytochemical compounds with high affinity to the FMDV 3C^pro^ active site and evaluated their antiviral activities using direct protease inhibition and cell-based assays. We demonstrated one of the mechanisms, in which the new candidate natural compounds inhibited FMDV by acting upon 3C^pro^. This inhibition effect may be true for other picornaviruses as the 3C^pro^ active site is highly conserved among viruses in this family.

## 2. Materials and Methods

### 2.1. Structure Modeling of FMDV 3C Protease and Validation

The crystal structures of FMDV 3C^pro^ were acquired from Research Collaboratory for Structural Bioinformatics (RCSB) Protein Data Bank (PDB). The available structures contained mutations of the catalytic residues (e.g., C95K, C163A, D84E) and active residues (e.g., C142S or C142L). Fitting of our 3C^pro^ sequence with the known 3C^pro^ structures was performed using a protein homology modeling server, SWISS-MODEL (https://swissmodel.expasy.org, accessed on 9 February 2021) [29]. The full-length 3C^pro^ amino acid sequences of an FMDV serotype A, topotype ASIA, lineage SEA-97, A/TAI/NP05/2016 (NP05) with accession number: MZ923645, were submitted for modelling with related protein structures by running against all available 3C^pro^ of FMDV serotypes A and O templates. The 3-D structural model of FMDV 3C^pro^ was generated. The model quality of the predicted protein structure was assessed and validated by quantitative model energy analysis (QMEAN) [30], QMEANDisCo scoring function [31], and MolProbity [32]. The FMDV 3C^pro^ structure was prepared to remove unwanted molecules, and corrected charges and protonations as previously described [33]. A total of 5789 phytochemical structure files were retrieved from Pubchem (https://pubchem.ncbi.nlm.nih.gov, accessed on 15 February 2021) [34], Phenol-Explorer (http://phenol-explorer.eu/compounds, accessed on 15 February 2021) [35], and SuperNatural II (http://bioinformatics.charite.de/supernatural, accessed on 15 February 2021) [36], respectively, to generate a library in sdf format for the following virtual screening process. Physicochemical properties and ADMET characteristics of the compounds including absorption, distribution, metabolism, excretion, and toxicity were analyzed using OSIRIS Data Warrior version 5.0 [37] and SwissADME server [38]. 

### 2.2. Virtual Screening of Phytochemical Flavonoids

Virtual screening was performed with AutoDock Vina [39], which was built into the PyRx suite version 0.9.8 to evaluate the ligand-protein complex [40]. The compounds in the library were firstly screened for blind docking on the entire protein surface by setting a grid box of 50 Å × 50 Å ×50 Å to assess the specificity of compounds towards the substrate-binding pocket of the FMDV 3C^pro^ active site. For the specific interaction, the compounds that were placed in the active site by the first screening were selected for the following focus docking. The grid box was centered at the catalytic triad with x = 50, y = 50, z = 66, and the dimension size was 22 Å × 22 Å × 22 Å, respectively. The compounds with binding affinity less than –6.0 kcal/mol from the focus docking were further selected for cell-based antiviral screening. 

### 2.3. Cells, Viruses, and Compounds

Baby Hamster Kidney (BHK−21) cells (ATCC^®^, Manassas, VA, USA) were maintained in a complete medium containing Minimum Essential Medium (MEM, Invitrogen™, Carlsbad, CA, USA), 10% fetal bovine serum (FBS, Invitrogen™, Carlsbad, CA, USA), 2 mM L-glutamine (Invitrogen™, Carlsbad, USA), and 1×Antibiotic-Antimycotic (Invitrogen™, Carlsbad, USA). FMDV serotype A (NP05) was propagated in BHK21 cells for 24 h. The virus stock was titrated, and the titer was calculated according to the Reed–Muench method [41] and reported as the median tissue culture infectious dose (TCID50) as described previously [42]. The virus stock with a titer of 1 × 10^7^ TCID50/mL was stored at −80 °C in aliquots. All experiments with the live viruses were conducted in a biosafety level 2 with an enhanced facility. All compounds were dissolved in DMSO to prepare 10 mM stock solutions for further assays.

### 2.4. Cytotoxicity Assay 

BHK−21 cells were seeded onto a 96-well plate at 1.8 × 10^4^ cells per well and incubated overnight. The spent media was replaced with fresh media containing serially diluted compounds (200, 100, 10, 1, 0.1, and 0.01 µM), and the compound-treated cells were further incubated at 37 °C with 5% CO_2_ for 24 h. The culture medium was discarded and washed once with phosphate buffer saline solution (PBS, pH 7.4) to remove the compound residues. The cytotoxicity was evaluated by MTS assay (Promega, Madison, WI, USA) according to the manufacturer’s instruction. The optical density was measured at 490 nm using a multi-mode reader (Synergy H1 Hybrid Multi-Mode Reader, BioTek^®^, Winooski, VT, USA), and the remaining attached cells were stained with 0.5% crystal violet. The data were calculated as the ratio between the blank subtracted treatment and control by the following equation:

$$\frac{\left[OD\;treated\;-OD\;cell\;control\right]}{\left[OD\;1\%dmso\;-\;OD\;cell\;control\right]}$$

Thus, the 50% cytotoxic concentration (CC50) value was defined as the compound concentration that reduced cell viability by 50%.

### 2.5. Antiviral Activity Assays (Prophylaxis, Viral Entry, and Post-Viral Entry)

**Figure 1:**
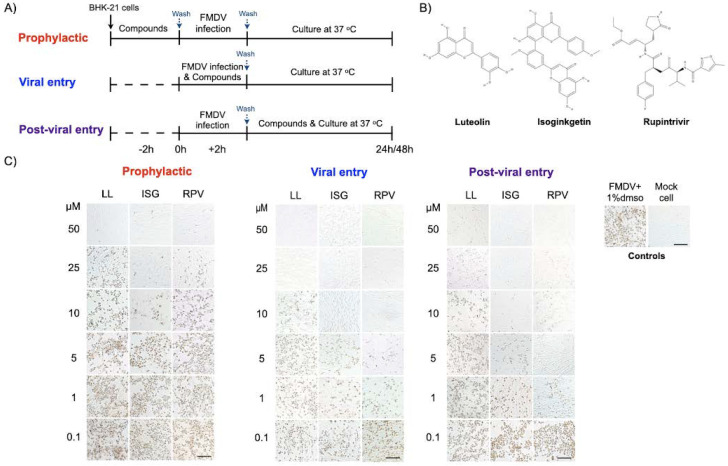
Antiviral activity of luteolin and isoginkgetin against FMDV in the cell-based assay. The compounds were treated at different times of FMDV infection, which were the prophylaxis (–2 h), viral entry (0 h), and post-viral entry (+2 h) experiments (**A**). The FMDV- and mock-infected BHK-21 cells in culture media with 1% DMSO were included as positive and negative virus controls, respectively (A, right panel). The 2-D structure of luteolin, isoginkgetin, and rupintrivir (**B**). The dose-dependent response was evaluated from the numbers of positive FMDV-infected cells using IPMA. Intensity and quantity of the dark brown cells directly reflects numbers of viral infected cells. The EC50 values were calculated using CellProfiler image analysis (**C**). LL (luteolin), ISG (isoginkgetin), and RPV (rupintrivir). The scale bar was 200 μm.

To evaluate the antiviral effect at various steps of viral infection, the BHK−21 cells were seeded at 1.8 × 10^4^ cells per well in a 96-well plate and incubated overnight. Various concentrations of each phytochemical compound were incubated with the FMDV and/or BHK−21 cells at different stages of viral infection including *prophylaxis*, *viral entry,* and *post-viral entry* (Figure 1A). The *prophylactic activity* of the compounds (–2 h) was evaluated by incubating the compounds on the cells for 2 h followed by viral inoculation. In this procedure, we examined the intracellular activities of the compounds against the virus; thus, cellular uptake of the tested compound was necessary. The cells were incubated with the serial diluted compounds at 37 °C for 2 h. Subsequently, the culture media with compounds was removed and the cells were washed once with PBS pH 7.4 (Sigma Aldrich^®^, St. Louis, MO, USA). The cells were then inoculated with FMDV (10 TCID50/well) at 37 °C for 24 h.

The *viral entry* experiment (at 0 h of virus inoculation, 0 h) aimed to examine how the compounds interfered with viral attachment. The virus at 10 TCID50 was mixed with serially diluted compounds to a final concentration of 100, 50, 20, 10, 5, 1, and 0.1 μM per well prior to incubation with the cells at 37 °C for 2 h. The virus-drug mixture was then replaced with fresh culture medium and the cells were incubated at 37 °C for 24 h. In the *post-viral entry* experiment (+2 h), we examined the effects of the compounds on the virus after the entry step. The cells were incubated with FMDV at 10 TCID50/well at 37 °C for 2 h for viral adsorption. Then, the cells were washed once with PBS pH 7.4 (Sigma Aldrich^®^, St. Louis, MO, USA) before incubation with serially diluted compounds as mentioned above at 37 °C for 24 h. Rupintrivir (3C^pro^ inhibitor; Sigma Aldrich^®^, St. Louis, MO, USA), ribavirin (a broad-spectrum antiviral drug; Sigma Aldrich^®^, St. Louis, MO, USA), and DMSO (non-inhibitor vehicle, Sigma Aldrich^®^, St. Louis, MO, USA) were used as positive and negative controls, respectively. In all experiments, viral reduction was further evaluated as described in the following sections.

### 2.6. Immunoperoxidase Monolayer Assay (IPMA) for FMDV Antigen Detection

The presenting FMDV antigens in infected cells with or without tested compounds were detected using IPMA as previously described [43]. Briefly, the infected BHK-21 cells were fixed with cold methanol at room temperature for 20 min and then washed with PBS with 0.1% Tween 20 (Sigma Aldrich^®^, St. Louis, MO, USA) (1 × PBST). The single-chain variable fragment with Fc fusion protein (scFv-Fc) specific to 3ABC of FMDV was used as the primary antibody for viral detection. The fixed cells were incubated with an optimum concentration of the primary antibody at 37 °C for 1 h, and then washed with 1 × PBST. Subsequently, the cells were incubated with protein G, HRP conjugate (dilution 1:1000, EMD Millipore corporation, Temecula, CA, USA) at 37 °C for 1 h. The antigen–antibody reaction was stained using DAB substrate (DAKO, Santa Clara, CA, USA) and the dark-brown color of viral-infected cells was observed under a phase-contrast inverted microscope (Olympus IX73, Tokyo, Japan). The cell images were recorded, and antiviral activity was determined by the decreased numbers of infected cells using CellProfiler software (version 4.1.3), with an open-source code of available algorithms (Board Institute, Cambridge, MA, USA; http://www.cellprofiler.org/index.htm, accessed on 9 May 2021) as previously described [44]. As a result, antiviral activity was reported as 50% effective concentration (EC50), which was analyzed using non-linear regression.

### 2.7. RT Real-Time PCR (RT-qPCR) for Viral Load Quantification

BHK−21 cells were seeded at 5.6 × 10^4^ cells/well onto a 48-well plate and incubated at 37 °C with 5% CO_2_ for 16–18 h. The cells were infected with FMDV at 10 TCID50 as described above, and virus yield was determined by the following RT-qPCR procedure. Total RNAs from FMDV-infected cells and supernatant with or without compounds and non-inhibitor vehicle were extracted using Direct-zol MiniPrep (Zymo Research Corporation, Tustin, CA, USA) following the manufacturer’s instruction. RNA quality was determined using a NanoDrop™ 2000 c Spectrophotometers (Thermo Fisher Scientific, Waltham, MA, USA). Complementary DNA (cDNA) was synthesized by RevertAid Reverse Transcriptase (Thermo Fisher Scientific, Waltham, MA, USA; 200 U/µL) in a 20-µL reaction containing 5X Reaction Buffer (250 mM Tris-HCl, 250 mM KCl, 20 mM MgCl2, 50 mM DTT), 10 mM dNTP, and 5 U of RNase H. The cDNA was subsequently used as the template in the following SYBR real-time PCR. 

For viral DNA copy quantification, a plasmid carrying FMDV 5′UTR was generated. Briefly, the 5′UTR was amplified using p0189 5′UTR [42] as the template. The primer sequences for PCR cloning are presented in Supplementary Materials Table S1. Then, the 5′UTR DNA fragment was inserted into the pGEM-T Easy plasmid (Promega, Madison, WI, USA). For absolute quantitation, the plasmid was 10-fold serially diluted with H_2_O to the concentrations ranging from 10^−2^ to 10^−7^ plasmid molecules/μL to generate a standard curve of the cycle threshold versus genomic copy numbers using SsoFast EvaGreen Supermix (Bio-Rad Laboratories, Hercule, CA, USA). The cycle condition was initial DNA denaturation at 95 °C for 30 s and 30 cycles of denaturation at 95 °C for 5 s and annealing plus extension at 60 °C for 5 s, followed by a melting curve analysis from 65 to 95 °C with a 0.5 °C increment as described previously [44]. Three biological and three technical replications were performed for each sample. The percentage of viral reductions was calculated relative to the viral control in the dose–response manner.

### 2.8. Construction of Plasmids for Intracellular Protease Assay

Plasmids carrying either a native 3ABCD ORF (p3ABCD) or a 3ABCD ORF containing mutated 3C^pro^ with Cys142Ser and Cys163Gly (pmu3ABCD) were generated as previously described [45] with slight modification. The primer sequences for PCR cloning are presented in Supplementary Materials Table S1. Plasmids pBIND-VP16 and pG5luc were purchased from Promega, Madison, WI, USA [46]. To produce pBIND_FMDV 3C^pro^ plasmids for use in the protease assay, the 3ABCD and mu3ABCD DNA fragments in p3ABCD and pmu3ABCD were subcloned into pBIND-VP16 plasmid (Promega, Madison, WI, USA) between a GAL4-binding domain and a VP16 activation domain at the *BamH* I and *Mlu* I sites, resulting in pBV_3ABCD and pBV_mu3ABCD, respectively. The pG5Luc containing the GAL4-binding site upstream of the firefly luciferase gene was used as a reporter system while pBV_3ABCD and pBV_mu3ABCD also carried the *Renilla* luciferase gene, which served as an internal luciferase control.

### 2.9. Protease Inhibition Activity Using Cell-Based Protease Assay

HEK 293T cells were seeded onto a 96-well plate and incubated overnight to yield 90% monolayers on the transfection day. One hundred nanograms of either pBV_3ABCD or pBV_mu3ABCD and 100 ng of pG5luc plasmid were mixed with 0.6 μL of Fugene^®^ HD (Promega, Madison, WI, USA). The DNA-Fugene^®^ HD mixture was gently overlaid on the cells before incubation at 37 °C with 5% CO_2_ for 2 h. Meanwhile, each compound was 10-fold serially diluted in Opi-MEM. Then, the transfection media was replaced with 100 µL of the diluted compound or 1% DMSO in the Opi-MEM with 2% FBS. After 16 h of transfection, the cells in each well were washed once with PBS and lysed with cell lysis buffer (Promega, Madison, WI, USA). Firefly and *Renilla* luminescence with signal stability were generated using the Dual-Glo Luciferase Assay System (Promega, Madison, WI, USA) following the manufacturer’s protocol. The luminescent levels were quantitated using a Synergy H1 Hybrid Multi-Mode Microplate Reader (BioTek, Winooski, VT, USA). The inhibition effect of each selected compound on FMDV 3C^pro^ activity was determined based on the ability of the compounds to inhibit the function of the wild-type 3C^pro^ expressed from pBV_3ABCD. The plasmid containing mutated 3C^pro^, pBV_mu3ABCD, was included as a protease negative control. The 50% inhibitory concentration (IC50) value was the compound concentration that increased the firefly/*Renilla* luminescent (Fluc/Rluc) ratio by 50% compared with the non-drug control. The IC50 of each compound was calculated based on the following formula:

$$\mathrm{IC}50=\frac{\mathrm{Fluc}/\mathrm{Rluc}\;\mathrm{sample}\;-\;\mathrm{Fluc}/\mathrm{Rluc}\;\mathrm{DMSO}}{\mathrm{Fluc}/\mathrm{Rluc}\;\mathrm{DMSO}}\times 100$$

### 2.10. Expression and Purification of Recombinant FMDV 3C Protease

The gene encoding FMDV 3C^pro^ was amplified using viral cDNA and cloned into pET16b vector (Merck KGaA, Darmstadt, Germany) by using PCR with the forward primer containing the *Nde*I cleavage sequence and reverse primer containing the *Xho*I cleavage sequence (Table S1). The recombinant FMDV 3C^pro^ plasmid was then transformed to *E. coli* DH5α-competent cells and the transformed cells were streaked on a Luria–Bertani (LB) agar plate containing 100 μg/mL ampicillin. Ampicillin-resistant colonies were selected from the agar plate and cultured in 5 mL of LB broth containing 100 μg/mL ampicillin overnight at 37 °C. The correct constructs were subsequently transformed to *E. coli* BL21 (DE3) for protein expression. Then, 5 mL of overnight culture of a single transformant were used to inoculate 500 mL of fresh LB medium containing 100 μg/mL ampicillin. The cells were grown to the appropriate optical density (OD600 = 0.6) and induced with 1 mM Isopropyl-β-D-1 thiogalactopyranoside. After 4–5 h, the cells were harvested by centrifugation at 7000× *g* for 15 min. 

The FMDV 3C^pro^ purification was conducted at 4 °C. The cell paste obtained from 1-L cell culture was suspended in 30 mL of phosphate-buffered saline (PBS). A French press instrument (AIM-AMINCO spectronic instruments; Cambridge Scientific Products, Watertown, MA, USA) was used to disrupt the cells at 12,000 psi. The lysis solution was centrifuged, and the debris was discarded. The supernatant of the cell extract was harvested by centrifugation and loaded onto a 10-ml Ni-NTA column equilibrated with PBS containing 5 mM imidazole. The column was washed with 5 mM imidazole followed by 30 mM imidazole-containing buffer. The His-tagged 3C^pro^ eluted with the buffer containing 300 mM imidazole was then subjected to further purification with gel-filtration chromatography on a Superose 12 10/300 GL column (GE Healthcare Inc., Princeton, NJ, USA) with PBS buffer. The His-tagged FMDV 3C^pro^ was dialyzed into a buffer containing 12 mM Tris-HCl (pH 7.5), 120 mM NaCl, 0.1 mM EDTA, and 2 mM DTT for storage at −80 °C. The enzyme concentrations used in all experiments were determined from the absorbance at 280 nm.

### 2.11. In Vitro Protease Inhibition Using FRET Assay 

The in vitro protease inhibition assay was performed following the previously described procedures using 96-well black plates [47]. Each well of the enzyme reaction mixture contained 100 μL of 1.2 μM FMDV 3C^pro^, 10 μM fluorogenic substrate peptide (Dabcyl-KTSAVLQSGFRKME-Edans) in a reaction buffer of 10 mM 2-(N-morpholino) ethanesulfonic acid (MES) buffer, pH 6.5. The various concentrations of each compound and the enzyme reaction mixture were incubated for 20 min at room temperature before the addition of the substrate peptide. Enhanced fluorescence due to cleavage of the fluorogenic substrate was recorded every 1 min for 20 min at 355 nm excitation and 538 nm emission using a fluorescence plate reader (BMG FLUOstar OPTIMA Microplate Reader, Ortenberg, Germany). The initial velocities of the inhibited reactions were plotted against the different inhibitor concentrations to yield the IC_50_ value by fitting with the following equation:


$$A\;\left(I\right)=A\left(0\right)\times \left\{1-\left[\frac{I}{\left(I+IC50\right)}\right]\right\}\;$$

In this equation, *A*(*I*) is the enzyme activity with inhibitor concentration I, *A*(0) is the enzyme activity without inhibitor, and *I* is the concentration of inhibitor. Data are expressed as mean ± SD values of three independent experiments.

### 2.12. Selectivity Index

The selectivity index (SI) of each phytochemical compound was determined as the ratio of CC50 and EC50. The CC50 values from the cytotoxicity assay were calculated depending on the decline of viable cells in the MTS assay, whereas the EC50 values were obtained from the reduction of the positive FMDV-infected cells in the presence of the tested compounds by IPMA. The IC50 values acquired from the cell-based protease assay were used to determine the potency of the potential FMDV 3C^pro^ inhibitors. The CC50, EC50, and IC50 values were calculated using a non-linear fitting curve following log10 transformation of the compound concentrations. The assays were carried out in BHK-21 and HEK293T cells, and the analyses were performed using GraphPad Prism version 8.0 (Prism, San Diego, CA, USA). 

## 3. Results

### 3.1. Homology Modeling and Virtual Screening of FMDV 3C^pro^

The three-dimensional structure of FMDV NP05 3C^pro^ was predicted by comparison with all available 3C protease PDBs. The NP05 3C^pro^ sequence matched with 527 templates and it was 96.24% and 95.17% identical to FMDV 3C^pro^ serotype A from PDB ID: 2WV4.pdb and PDB ID: 2J92.pdb, respectively, in the 50 top-ranked templates (Figure S1). The distribution of the protein backbone dihedral angles in the homology model was in the Ramachandran-favored region with scoring at 95.00%. The GMQE (Global Model Quality Estimate) score for template selection was 0.87, suggesting good alignment between the target and template sequences as well as the structures. The predicted model was comparable to the PDB structure with a QMEAN score of –0.72 and QMEANDisCo score of 0.86 ± 0.06, respectively, which reflected the prediction reliability. Our prediction model did not demonstrate a major difference from the 3-D structure created using PDB ID: 2WV4 [12] as the FMDV 3C^pro^ template. The structure quality of the protein model is given in Figure S1.

We utilized the structure-based virtual screening approach to search for FMDV 3C^pro^ inhibitors. In total, 100 of 5789 ligands obtained from the first hit-list were qualified for subsequent secondary virtual screening. The lowest binding affinities within the second hit-list ranged from –6.5 to –7.5 kcal/mol. The binding energy between the 3C^pro^ and the two drug controls, ribavirin and rupintrivir, was −5.9 and −6.3 kcal/mol, respectively (Figure S2). As a result, 23 compounds were defined as ‘active’ by showing a stronger binding in the catalytic pocket. The physicochemical properties and their binding affinities are presented in Table S2. The significant results are discussed in the following sections.

### 3.2. Cytotoxicity and Antiviral Activity of the Phytochemical Compounds

The 23 phytochemical compounds that passed the secondary virtual screening were subsequently examined for their inhibitory effects on FMDV replication and cytotoxicity to BHK-21 cells in a cell-based assay. Most compounds possessed high CC50 values (>100 μM). However, amentoflavone, GCG, silibinin, and ZINC4026679 were substantially toxic to BHK-21 cells, with CC50 of 42.56, 56.25, 45.10, and 37.89 µM, respectively. We further tested the antiviral activities of the compounds and determined the effect of each compound on the viral life cycle. The compounds were examined by three different treatment procedures, in which the compounds were incubated with cells before or after FMDV infection (Figure 1A). The spectrum of virus-infected cells was determined by IPMA, in which viral proteins were stained with brown color (Figure 1C). To identify the high effective inhibitory effects, we treated the non-toxic compounds at the maximum concentration (100 μM), and the mild toxic compounds at 50 μM for 48 h. The non-drug treatments were included as the control groups. The result suggested that luteolin and isoginkgetin could completely inhibit the virus either during adsorption (*viral entry*) or post infection (*post-viral entry*), whereas apigenin, methyl-luteolin, and quercetin 7-rhamnoside (EC50 = 75–100 μM) had low antiviral activities.

**Figure 2:**
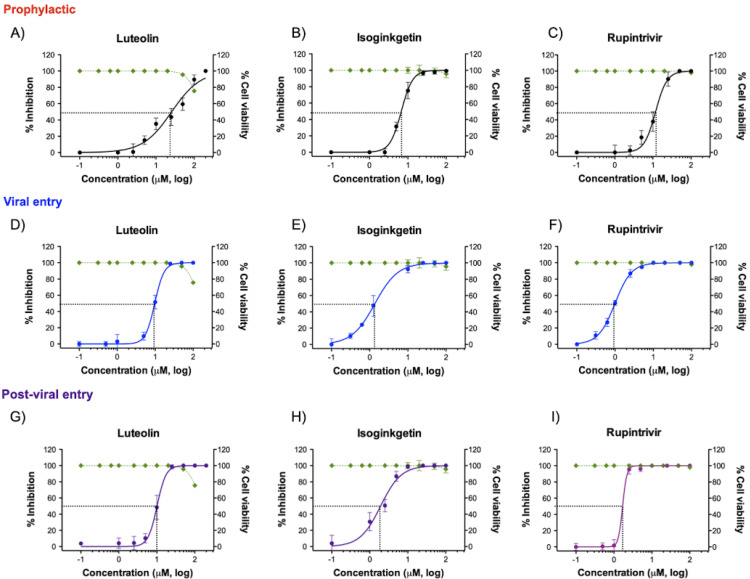
Dose–response curves between the effects of luteolin and isoginkgetin on FMDV inhibition and their cytotoxic effects on BHK-21 cells were established. Antiviral activities of the phytochemical compounds in the *prophylaxis* (**A**–**C**), *viral entry* (**D**–**F**), and *post-viral entry* (**G**–**I**) experiments were determined at different times of infections. The FMDV-infected cells were treated with different compound concentrations as depicted in Figure 1A and the numbers of FMDV-infected cells were determined by IPMA. The viability of the treated cells was also measured and compared to that of the cell control with 1% DMSO (non-inhibitor control) using the MTS assay (green line). Rupintrivir was used as a positive antiprotease drug control. All experiments were carried out in triplicates.

To further explore the inhibition mechanisms of the two flavonoids against FMDV infection, we performed the compound treatment at three different time periods (with 2-h intervals) in the cell-based assays (Figure 1A). The results showed that luteolin (EC50 = 9.73 ± 0.94 μM; SI > 10.27) and isoginkgetin (EC50 = 2.01 ± 0.07 μM; SI > 49.75) could interfere with viral adsorption with a greater effect than ribavirin. The antiviral activities of isoginkgetin and rupintrivir were comparable in the *viral entry* condition (Figure 1C and Table 1). These two compounds also exhibited strong inhibition by blocking at the *post-viral entry* stage with high SI values (luteolin, EC50 = 10.00 ± 0.98 μM; SI > 10 and isoginkgetin EC50 = 1.93 ± 0.21 μM; SI > 51.81). To evaluate the cellular uptake and prophylactic activity of the tested compounds, we pre-treated the cells with the test compounds for 2 h, and then washed the compound residues before FMDV infection. In this procedure, a compound can protect the host cells only when it penetrates the cell membrane and is still active intracellularly. We found that luteolin and isoginkgetin also had *prophylactic activity*, but were slightly less effective than the *viral* and *post-viral entry* conditions (luteolin, EC50 = 25.83 ± 1.29 μM; SI > 3.87 and isoginkgetin EC50 = 6.76 ± 0.80 μM; SI > 14.79). Our cell-based assays showed that anti-FMDV infections of luteolin and isoginkgetin could arise mainly from interference with the viral life cycle including polyprotein processing. In addition, the antiviral actions of both drugs were dose dependent as shown by the dose–response curves (Figure 2).

### 3.3. Viral Quantification by RT-qPCR

**Figure 3:**
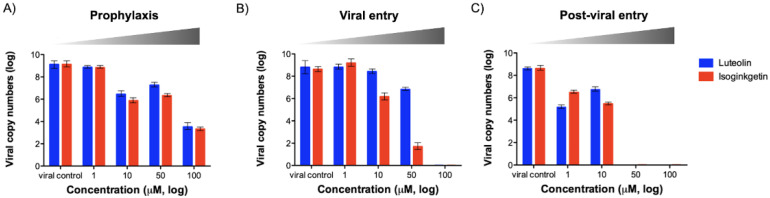
Dose-dependent inhibition of luteolin and isoginkgetin on FMDV replication as examined by RT-qPCR. The antiviral activities of each compound were determined by the reduction of viral copy numbers in the FMDV-infected cells compared to that of the virus control with 1% DMSO (non-inhibitor control). *Prophylaxis* (**A**), *viral entry* (**B**), and *post-viral entry* (**C**) experiments were designed as shown in Figure 1A. The data are reported as means and SD of viral copy numbers from triplicate experiments.

The RT-qPCR-based viral reduction assay was performed to quantify the copy numbers of both intracellular and extracellular viral nucleic acids (Figure 3). The two candidate compounds at 100 µM markedly reduced the viral yields at 24 h post treatment in the *viral* and *post-viral entry* assays. Additionally, both phytochemical compounds at a high dose (100 µM) could decrease more than five log of the viral nucleic acids in the *prophylactic* assay. This indicates that the compounds could penetrate the cells, but the antiviral activities might decrease during the pre-incubation step (Figure 3A). The RT-qPCR and IPMA results were in accordance and confirmed that luteolin and isoginkgetin affected FMDV infection in a dose-dependent manner.

### 3.4. Evaluation of FMDV 3C^pro^ Inhibitors Using Cell-Based Protease Assay

To evaluate the ability of luteolin and isoginkgetin to inhibit the protease activity of FMDV 3C^pro^, we developed a cell-based protease assay. In this assay, non-processed 3ABCD polyprotein bridges the VP16 activation domain (AD) to the GAL-4-binding domain (BD). GAL-4 BD has a binding site on the plasmid DNA near the firefly luciferase (Fluc) promotor. Binding of GAL-4 BD to DNA brings VP16 AD in the proximity of the promotor to induce Fluc expression. Without an inhibitor, the expressed 3C^pro^ functions properly and cuts the polyprotein 3ABCD at the cleavage sites between 3A|3B, 3B|3C and 3C|3D into single functional proteins, and thus separates VP16 AD from GAL-4 BD, resulting in no luciferase expression. Once 3C^pro^ is inactivated by an inhibitor, the polyprotein is not processed and allows expression of the luciferase gene. In the previous experiments, 23 phytochemical compounds could pass the initial filtering by double virtual screening based on the 3C^pro^ 3-D structure and five of them were effective against FMDV in the cell-based assay. These five compounds were further tested for their inhibitory effects on the viral protease using the intracellular protease assay.

**Figure 4:**
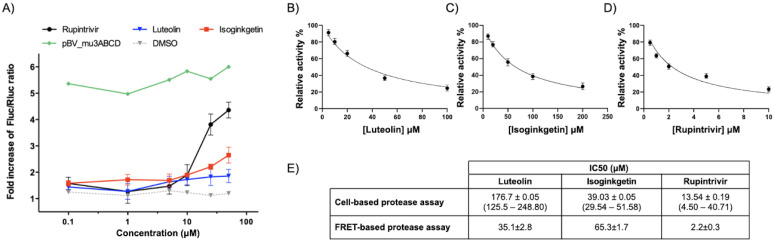
Cell-based and FRET-based protease assay used to evaluate the anti-FMDV 3C^pro^ activities of luteolin and isoginkgetin. pBV_3ABCD containing a gene encoding for the FMDV-3ABCD GAL4-binding domain and VP16 activation domain, and pG5luc reporter plasmid with the GAL4-binding site were co-transfected into HEK293T cells prior to incubating with the 10-fold serially diluted compounds. The firefly (Fluc) and *Renilla* (Rluc) luciferase levels generated from each treatment was measured and reported as Fluc/Rluc ratios (**A**). The IC50 values of luteolin (**B**), isoginkgetin (**C**), and rupintrivir (**D**) determined by the FRET-based protease assay were measured to be 35.1 ± 2.8, 65.3 ± 1.7, and 2.2 ± 0.3 μM, respectively, based on the inhibitor concentration-dependent curves. The IC50 values obtained from two different protease assay methods were summarized in (**E**).

Among them, luteolin and isoginkgetin, which showed high antiviral efficacy against the FMDV life cycle, could also block the 3C^pro^ activity. The 50% inhibition concentration (IC50) value of luteolin was 176.7 ± 0.05 μM with a 1.86-fold increase of the FLuc over *Renilla* luciferase (Rluc) ratio. The IC50 of isoginkgetin was 39.03 ± 0.05 μM with a 2.65-fold increase of the luciferase ratio. Apigenin, quercetin 7-rhamnoside, and 7-O-methyl-luteolin were less effective and could inhibit FMDV 3C^pro^ activity at a dose greater than 200 μM. Rupintrivir, an HRV 3C^pro^ inhibitor, was included as a protease inhibitor control and its IC50 value was 13.54 ± 0.19 μM with a 4.27-fold increased signal. The fold increase of the Fluc/Rluc ratios and IC50 values are shown in Figure 4A. DMSO was a non-drug control while pBV_mu3ABCD was included as the inactivated 3C^pro^ control.

### 3.5. Protease Inhibition Using FRET Assay

The peptide KIIAPAKQ↓LLNFDLLK (↓ represents the cleavage site) corresponding to the viral VP1/2A junction has been reported as the best substrate for FMDV 3C^pro^ [9]. In this study, we found that the fluorogenic substrate (Dabcyl-KTSAVLQSGFRKME-Edans) of SARS-CoV 3CL^pro^ [47] with the same P1-Gln can be used as a substrate for the FMDV 3C^pro^ inhibition assay. The potency of the two candidate compounds (luteolin and isoginkgetin) screened by the cell-based protease assay was further confirmed by the FRET-based protease assay. Recombinant FMDV 3C^pro^ was incubated with varying concentrations of luteolin, isoginkgetin, and rupintrivir (reference compound). After adding the fluorogenic substrate, the initial velocity of the hydrolysis reaction was recorded by monitoring the increase of fluorescence. The calculated IC50 values of luteolin, isoginkgetin, and rupintrivir were 35.1 ± 2.8, 65.3 ± 1.7, and 2.2 ± 0.3 μM, respectively (Figure 4B–D), demonstrating the antiviral effects of luteolin and isoginkgetin against FMDV via targeting of the 3C proteases.

### 3.6. Interaction of Luteolin and Isoginkgetin with FMDV 3C^pro^

**Figure 5:**
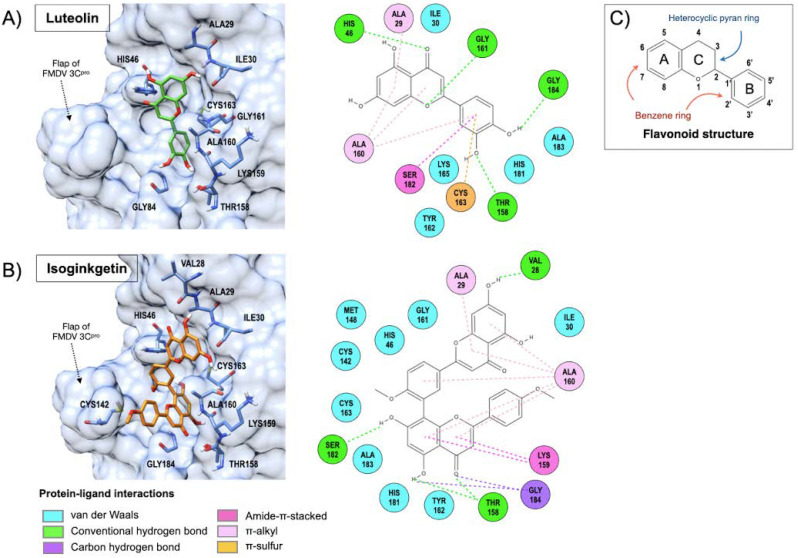
Molecular docking of FMDV 3C^pro^ and protein–ligand interactions of luteolin (**A**) and isoginkgetin (**B**) with the binding pocket of FMDV 3C^pro^. The illustration colors for the protein-ligand bonds and interactions are presented at the bottom of Figure (**B**). The basic core structure of flavonoid contains two benzene rings (A and B rings) connected by a heterocyclic pyran ring (C ring) as shown in (**C**). Luteolin reacted to the catalytic residues, His46 and Cys163, whereas isoginkgetin showed van der Waals interaction with His46 and Cys163 as well as the Cys142 flap. The color circle of the amino acids indicates the interactive residues of the 3C^pro^ and bond types as depicted at the bottom of the left panel.

Among the five phytochemical compounds, both cell-based assays and the FRET-based protease assay showed that luteolin and isoginkgetin were potent antiviral compounds. In addition, the molecular docking revealed that both compounds fitted well in the 3C^pro^-binding pocket with high affinities (Figure 5). The protein–ligand interactions demonstrated that both compounds associated with key residues in the enzymatic active sites as depicted in Figure 5. Luteolin was well placed in the FMDV 3C^pro^-binding pocket (Figure 5A), where His46 of the catalytic triad was linked to a carbonyl group of the heterocyclic pyran ring with hydrogen bonding and Cys163 reacted with the benzene ring with π–sulfur interaction. Other interactions with luteolin included π-alkyl (Ala29 and Ala160), amide-π stacked (Ser182), and hydrogen bonds (Thr158 with C3′ hydroxyl, Gly161 with oxygen atom of heterocyclic pyran ring, and Gly184 with C4′ hydroxyl). Isoginkgetin interacted with His46 and Cys163 of the catalytic triad as well as the Cys142 flap via van der Waals interactions (Figure 5B). Two flavonoid backbones of isoginkgetin connected with amino acids by amide-π stacked (Lys159) and π-alkyl (Ala29 and Ala160) interactions, and hydrogen bonds (the remaining residues).

The molecular docking results suggested that luteolin interacted with the key residues of the catalytic triad mainly via π-sulfur and hydrogen bonding. Isoginkgetin could react to the catalytic residues with van der Walls interactions and the structure mostly buried in the catalytic pocket of FMDV 3C^pro^. The orientations of both compounds in the enzyme active site could interfere with the basic residue (His46) polarization, which is normally required for nucleophile residue (Cys163) activation in the catalytic triad charge-relay system and affects the C142 flap stability of FMDV 3C^pro^.

## 4. Discussion

The homology modeling revealed that both the amino acid sequence and three-dimension structure of 3C^pro^ are similar to those of FMDV 3C^pro^ available in the RCSB PDB database. The precision of the predicted model was adequate for virtual screening as determined by the QMEAN Z-score and Ramachandran plots. We showed that virtual screening based on the FMDV 3C^pro^ structure could accelerate the search for potent antiviral agents by double filtering 23 out of 5789 phytochemical compounds readily for successive time-consuming and high-cost selection processes. The results of the cell-based antiviral assay revealed promising anti-FMDV activities of luteolin and isoginkgetin, confirming the reliability of the virtual screening process. Moreover, the EC50 values obtained from our cell-based assay were reliable as the FMDV-infected cells were detected by IPMA. This method was more specific than observing viral CPE, in which discrimination between regular and viral-induced dead cells could be problematic. In addition, the antiviral properties of luteolin and isoginkgetin were validated by viral RNA reduction using real-time qPCR. Therefore, the SI values of luteolin and isoginkgetin with the range of 10.00–51.81 μM in *viral* and *post-viral entry* experiments were greater than 4, which is considered as potent antiviral agents [48].

We further evaluated the protease inhibitory effect of both phytochemical compounds by the cell-based and FRET-based protease assays. The IC50 values calculated by the two assays varied (Figure 4E), which could be caused by the diverse analytical methods. Moreover, in our study, higher concentrations of luteolin and isoginkgetin might be required to directly inhibit the FMDV 3C^pro^ protease activity in the cell-based and FRET-based assays, compared to the doses that could block viral replication in the antiviral cell-based assay. This finding is consistent with other studies in picornaviruses [28,48]. They determined the protease activity of rupintrivir, an HRV 3C^pro^ inhibitor, and found that the IC50 was also higher than the EC50.

The interest in the benefits of flavonoids from natural sources on human health has been increasing for a decade. Flavonoids demonstrated potential antiviral activities against a wide range of DNA and RNA viruses including picornaviruses by several mechanisms (reviewed in [22]). Flavonoids can block viral attachment and entry, replication, translation, and polyprotein processing as well as interfere with the host factors to prevent viral release and spread to other cells. Flavonoids comprise various phenolic structures (Figure 5), the basic backbone of which contains 15 carbons of 2 benzene rings linked by a heterocyclic pyran ring [49]. The flavonoids are classified into several classes, such as flavone (e.g., luteolin) and biflavone (e.g., isoginkgetin). Luteolin, a phytochemical flavonoid, has numerous therapeutic properties, such as anti-inflammatory, antioxidant, anti-tumor, antimicrobial, and antiviral activities [50]. It was found that luteolin could counteract several viruses including Japanese B encephalitis virus (JEV) [51], Epstein–Barr virus (EBV) [52], and human immunodeficiency virus (HIV) (by targeting Tat protein) [53]. Furthermore, luteolin appeared to be a potential antiviral agent against a number of picornaviruses, including Coxsackievirus A16 (CVA16), CVB3, and enterovirus A71 (EV71), by inhibiting RNA replication at the post-viral entry stage [23,25,54] in accordance with our results. Other flavonoids, such as rutin, fisetin, and quercetin, have been shown to inhibit EV71 3C^pro^ activity [27,28].

In addition, luteoloside, a plant glycosyloxyflavone, which is a group of luteolin derivatives consisting of a beta-D-glucopyranosyl moiety substitution at the C7 hydroxyl group, could block EV71 3C^pro^ activity [55]. Moreover, luteolin contains an oxygen and a carbon double bond in the heterocyclic pyran ring (4-CO groups of C ring), and hydroxyl groups at 3-OH of the C ring or 5-OH of the A ring as well as 3′- and 4′-OH of the B ring. These moieties have free radical scavenging activity and can chelate metal ions [56]. The basic molecular mechanism has been recently proposed, stating that the hydroxyl groups of the benzene ring (B ring) of natural flavonoids could directly react with the S1 pocket of serine protease [57].

Isoginkgetin is a well-known phytochemical biflavone from *Ginkgo biloba* and its antiviral activity has been reported previously. Isoginkgetin-related groups with the isoginkgetin-like structure, such as ginkgetin, also showed an antiviral effect on herpes simplex virus types I and II [58]. In addition, both isoginkgetin and luteolin have been shown to effectively inhibit other proteases, including thrombin, a serine protease, by the interaction between hydroxyl groups of isoginkgetin and the protease via hydrogen bonding and salt bridges [59]. Isoginkgetin was previously known as a pre-mRNA splicing inhibitor [60] and it could elevate Bax proapoptotic protein and caspase-3 expression in apoptotic cells [61]. Recently, isoginkgetin has been shown to inhibit the protease activity of proteasome [62]. However, the antiviral mechanisms of isoginkgetin have not been completely elucidated yet. 

## 5. Conclusions

We utilized virtual screening based on the structure of the viral major protease, 3C^pro^, to filter potential anti-FMDV inhibitors from phytochemical compound libraries. Among the 5789 tested molecules, two flavonoids, luteolin and isoginkgetin, demonstrated a high potent negative effect on the FMDV life cycle by blocking the 3C^pro^ activity. The molecular docking revealed a good binding affinity of both compounds and FMDV 3C^pro^, which strengthened our findings by the cell-based and FRET-based protease assays. As both phytochemical flavonoids presented negative effects on both DNA and RNA viruses in several studies, they could become promising board-spectrum antiviral agents effective against FMDV and other viruses.

## Figures and Tables

**Table 1:** Binding affinity to FMDV 3C^pro^, cytotoxicity to BHK-21 cells, and anti-FMDV activity of phytochemical compounds.

Compounds	Binding Affinity (kcal/mol)	Cell-Based Assay			
		Cytotoxicity (CC50 ^a^; μM)	Prophylaxis (EC50 ^b^; μM)	Viral (EC50; μM)	Post-Viral (EC50; μM)
Luteolin (ChemFaces)	−7.0	>100 ^e^	25.83 ± 1.29	9.73 ± 0.94	10.00 ± 0.98
Isoginkgetin (TargetMol)	−7.2	>100	6.76 ± 0.80	2.01 ± 0.07	1.93 ± 0.21
Apigenin (Vitas-M Laboratory)	−7.0	68.73 ± 4.10	>100	75–100	75–100
Quercetin 7-rhamnoside (ChemFaces)	−6.8	>100	>100	>100	75–100
7-O-Methyl luteolin (ChemFaces)	−6.3	>100	>100	75–100	75–100
Ribavirin (Sigma Aldrich) ^c^	ND	>100	283.90 ± 2.30	41.80 ± 1.58	133.30 ± 2.03
Rupintrivir ^d^ (Sigma Aldrich)	ND	>100	11.79 ± 1.03	1.99 ± 0.01	1.688 ± 0.19

^a^ CC50: compound concentration required to reduce cell viability by 50% as determined by MTS assay. ^b^ EC50: compound concentration required to effectively protect 50% of the units from virus infection as determined by IPMA. ^c^ Ribavirin (a broad-spectrum antiviral drug) and ^d^ rupintrivir (an HRV protease inhibitor) were included as drug controls. Values represent the means ± SD of three independent experiments.

## Data Availability

No new data was created or analyzed in this study. Data sharing is not applicable to this article.

## Supplementary Materials

The following are available online at https://www.mdpi.com/article/10.3390/v13112118/s1, Figure S1: The details of protein model quality, Figure S2: Binding affinities and the interaction of FMDV 3Cpro with ribavirin (A) and rupintrivir (B), Table S1: Sequences of the primers used for FMDV 3C^pro^ cloning, Table S2: Top twenty-three phytochemical compounds in this study demonstrating the binding affinities to FMDV 3C^pro^ and their physicochemical properties.
