# Supplementary material for: Natural Phytochemicals, Luteolin and Isoginkgetin, Inhibit 3C Protease and Infection of FMDV, In Silico and In Vitro
Source: Viruses. 2021 Oct 21;13(11):2118. doi: 10.3390/v13112118 (PMC8625466; doi:10.3390/v13112118)
Supplement: Supplementary file 1 [file viruses-13-02118-s001.zip › Supplementary Table S1.pdf]

SUPPLEMENT DATA

SUPPLEMENTARY TABLE

Supplementary Table S1: Sequences of the primers used for FMDV gene cloning.

Plasmids	Primers	Sequences from 5' to 3'
pFMDV_5'UTR	Forward	CTGTTGCTTCGTAGCGGAGC
	Reverse	TCGCGTGTTACCTCGGGGTACC
p3ABCD/	Forward	TAGAGGATCCTGAACTCCGAGCCCGCCAAACC
pmu3ABCD	Reverse	TGACGCGTTGACGTGCACGCGCTCTTCCACATCTCTGG
		TGTCAACGATCAACCCCTCGTGGTGTGGT
pFMDV_3C^{pro}	Forward	5'- GGAATTCCATATGAGTGGTGCACCCCCGAC-3'
	Reverse	5'- CCGCTCGAGTTATTCGTGGTGTGGTTCAGGG-3'

Note: Restriction sites are underlined
