# Supplementary figures and images for: Natural Phytochemicals, Luteolin and Isoginkgetin, Inhibit 3C Protease and Infection of FMDV, In Silico and In Vitro
Source: Viruses. 2021 Oct 21;13(11):2118. doi: 10.3390/v13112118 (PMC8625466; doi:10.3390/v13112118)

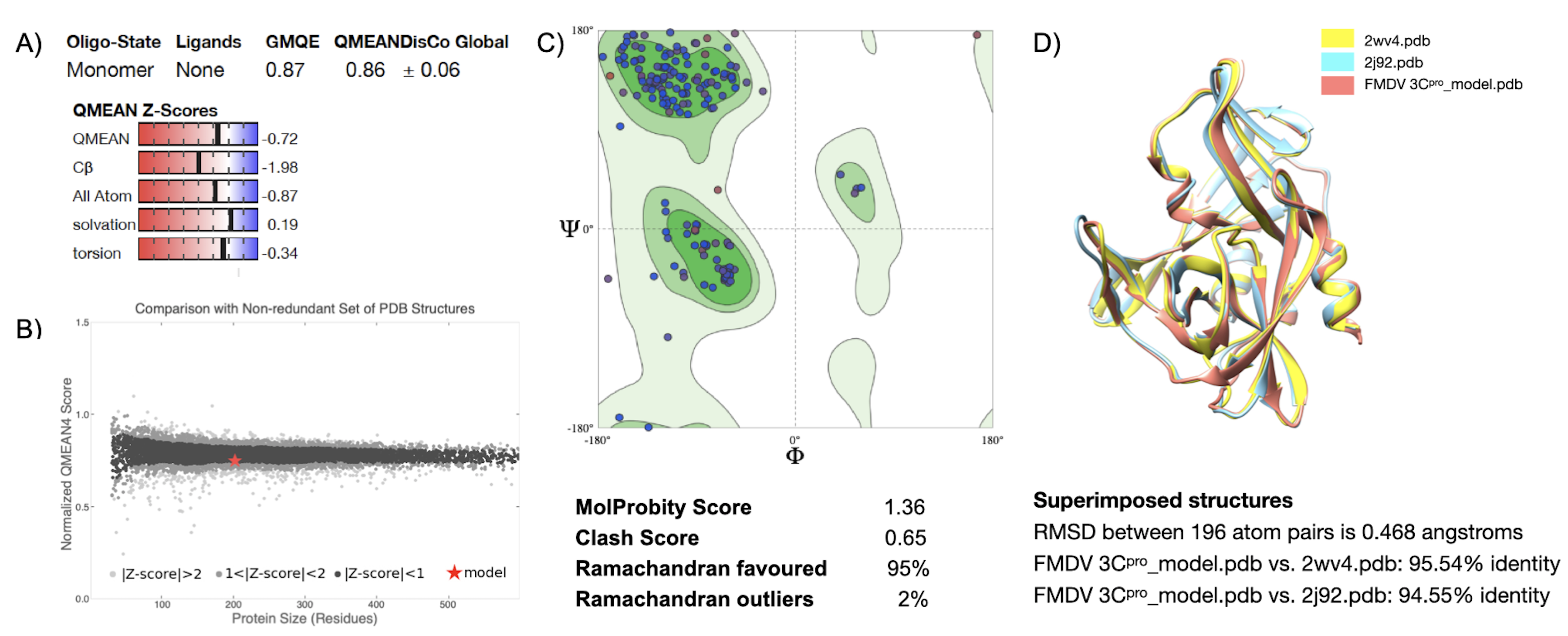

Supplement: Supplementary file 1 [file viruses-13-02118-s001.zip › Figure S1_edit.tiff]

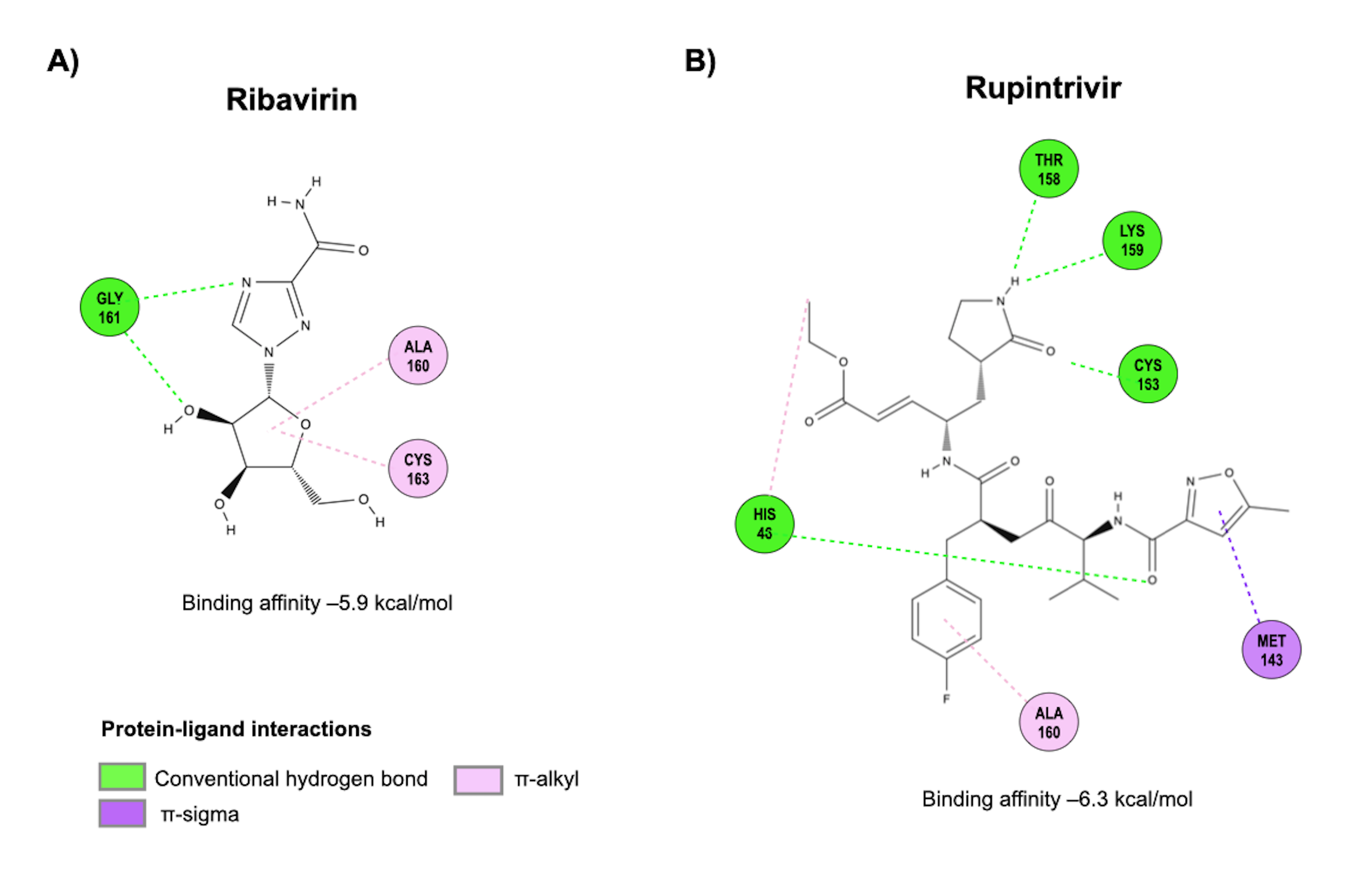

Supplement: Supplementary file 1 [file viruses-13-02118-s001.zip › Figure S2.tiff]
